# Targeting biomolecular condensates: beyond dissolution

**DOI:** 10.1186/s12915-026-02538-2

**Published:** 2026-02-02

**Authors:** Cassio Fleming, Jurian Schuijers

**Affiliations:** 1 Center for Molecular Medicine, University Medical Center Utrecht, Utrecht, 3584 CG the Netherlands; 2 Oncode Institute, Utrecht, 3721 AL the Netherlands

**Keywords:** Biomolecular condensates, Molecular therapeutics, Drug discovery

## Abstract

Biomolecular condensates control key cellular processes, from gene expression to signal transduction, by organizing molecules through selective compartmentalization. Increasing evidence links their dysregulation to cancer, neurodegeneration, and other diseases, positioning condensates as promising therapeutic targets. This review explores emerging strategies that go beyond dissolving pathological condensates, including approaches that induce, redirect, or reprogram their dynamics, composition, and physical state. Rather than inhibiting individual proteins, these interventions reshape the cellular organization itself. By targeting the material and functional properties of condensates, such strategies offer a new conceptual framework for therapeutic design in complex, dysregulated biological systems.

## Condensates as emerging drug targets

The intracellular space is an exceedingly crowded environment with proteins and nucleic acids reaching concentrations of up to 400 mg/mL, accounting for approximately 20–40% of the total cell volume [1]. Despite this, a strict organization of biochemical reactions needs to be maintained. Subcellular compartmentalization is a key organizational strategy that is often achieved through membrane-bound organelles. However, cells also rely on membraneless compartments, known as biomolecular condensates, which form through multivalent interactions. These interactions can arise from different molecular features, such as repeated domains and intrinsically disordered regions, each of which can promote dynamic and reversible molecular organization [2]. Many essential processes such as signaling [3], transcription [4], DNA repair [5], and RNA processing [6] have been associated with biomolecular condensates. Aberrant condensate formation or regulation has been linked to diverse disease states, including cancer, neurodegeneration, and viral infection (Table 1) [80]. Although the precise functional contributions of condensation remain under active investigation, this framework provides a new way to study how molecules organize intracellularly and offers new avenues for therapeutic intervention.

**Table 1:** Disease-associated biomolecular condensates. Condensate annotations were curated using the CD-CODE database [7]

Disease	Condensate	Protein	Reference(s)
**Cancer**	Cajal body	UHMK1	[8]
	Germ granules	DDX4	[9]
	Nuclear speckles	SIRT6	[10]
	Nuclear speckles	ZNF32	[11]
	Nucleolus	NONO	[12]
	Nucleolus	NPM1	[13, 14]
	Nucleolus	NPM1	[15]
	P bodies	EDC3	[16]
	P bodies	PJA2	[17]
	P bodies	RAS	[18]
	P bodies	UBAP2L	[19]
	P bodies	YAP	[20]
	PML bodies	PML	[21]
	Stress granules	ATF5	[22]
	Stress granules	BCR-ABL	[23]
	Stress granules	COX-2	[24]
	Stress granules	cPLA2	[25]
	Stress granules	G3BP1	[26, 27]
	Stress granules	G3BP2	[28]
	Stress granules	LIMK1	[29]
	Stress granules	P53	[30]
	Stress granules	PTEN	[31]
	Stress granules	RACK1	[32]
	Stress granules	RIOK1	[33]
	Stress granules	SRPK2	[34]
	Stress granules	TBK1	[35]
	Stress granules	UBAP2L	[36]
	Transcriptional condensates	ELM4-ALK	[37]
	Transcriptional condensates	EWS-FLI1	[38]
	Transcriptional condensates	LSD1	[39]
	Transcriptional condensates	SP1	[40]
	Transcriptional condensates	YAP	[41]
	Transcriptional condensates	ZHX2	[42]
	Transcriptional condensates	*β*-Catenin	[43]
**Metabolic dysfunction**	Paraspeckles	NONO	[44]
	Stress granules	G3BP1	[45]
**Neurodegenerative disease**	A bodies	cMyc	[46]
	Cajal body	VRK1	[47]
	P bodies	PSM8	[48]
	Stress granules	ALAVL4	[49]
	Stress granules	ATXN2	[50]
	Stress granules	C9ORF72	[51]
	Stress granules	FUS	[52]
	Stress granules	NUP107	[53]
	Stress granules	SYK	[54]
	Stress granules	TDP-43	[55–58]
	Stress granules	TIA1	[59]
	Transcriptional condensates	HAS1	[60]
**Viral infection**	Negri body	Viral proteins	[61]
	P bodies	Viral proteins	[62]
	P bodies	XRN1	[63]
	Stress granules	DDX17	[64]
	Stress granules	eIF2α	[65, 66]
	Stress granules	G3BP1	[67–75]
	Stress granules	Viral proteins	[76, 77]
	Transcriptional condensates	Viral proteins	[78, 79]

Therapeutic strategies targeting condensates focus on modulating their behavior by altering their formation, stability, localization, or material properties. Compounds that modify condensates in any of these ways have been termed “condensate-modifying drugs” (c-mods) and can act through diverse mechanisms beyond dissolving aberrant assemblies, for example, by inducing the formation of beneficial condensates, redirecting their localization, or changing their physical state [81]. Importantly, c-mods may enable therapeutic interventions targeting proteins traditionally considered “undruggable,” provided that they function within condensate compartments. Rather than targeting individual proteins, this approach seeks to reprogram entire condensate structures to shift cellular behavior, with early applications in cancer and neurodegeneration [82].

Among the many processes organized by condensates, signal transduction plays an important role in disease. In the Wnt pathway, key signaling proteins such as Dishevelled (Dvl) [83, 84], Axin [85, 86], and *β*-catenin [87, 88] form condensates that help coordinate pathway activity. Aberrant regulation of these assemblies can lead to sustained oncogenic signaling and has been linked to cancer. Similarly, the Hippo pathway relies on phase separation of the transcriptional coactivators YAP and TAZ [89], whose condensate-dependent activity contributes to tumorigenesis when misregulated [90]. In response to stress, cells form stress granules, transient condensates, which store untranslated mRNAs and RNA-binding proteins to regulate protein synthesis and promote recovery [91], but persistent or altered stress granule states are associated with neurodegenerative disease [92]. The innate immune sensor cGAS also forms condensates upon binding cytosolic DNA, amplifying STING signaling and inducing interferon production, a process implicated in autoinflammatory disease and cancer immunity [93]. These examples demonstrate the broad roles of condensates in intracellular signaling and how their dysregulation is linked to diverse disease contexts (Table 1).

**Fig. 1:**
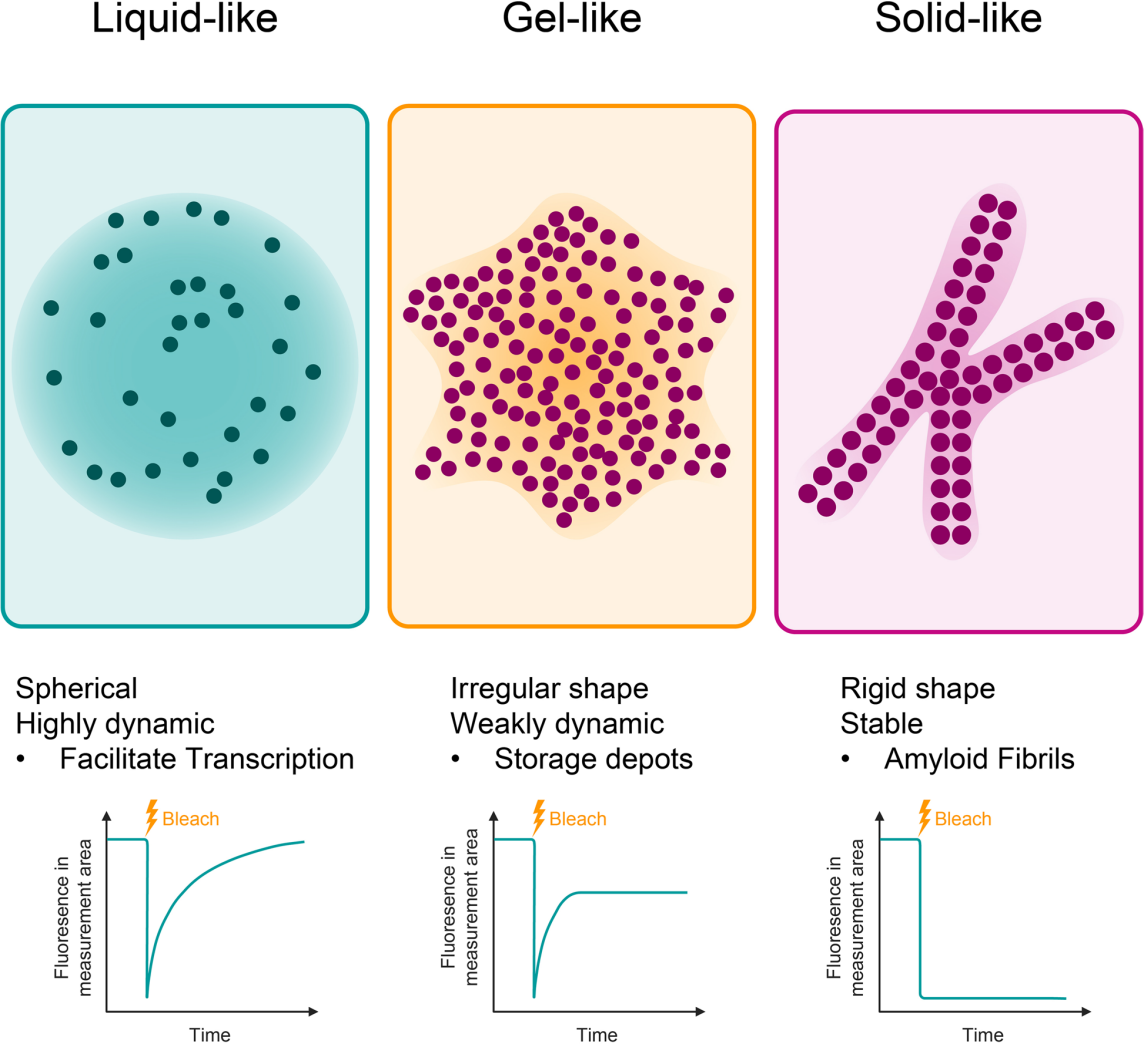
Phase transitions of biomolecular condensates. Top: Schematic representations of condensates in liquid-like, gel-like, and solid-like states, highlighting characteristic morphology and dynamics. Bottom: Schematic fluorescence recovery after photobleaching (FRAP) curves to illustrate expected dynamics: full recovery in liquid-like, partial in gel-like, and no recovery in solid-like states

Biomolecular condensates can transition between liquid-like, gel-like, and solid-like states, and these physical properties directly impact their biological function [94]. Liquid-like condensates are dynamic and reversible, allowing the rapid exchange of components with the surrounding environment. In contrast, gel-like condensates exhibit slower internal rearrangement and reduced molecular mobility. Solid-like condensates are more rigid and are often associated with pathological aggregation [95] (Fig. 1). Increasing evidence suggests that tuning the physical state of condensates may offer a way to control signaling output. In this review, we explore how the modification or induction of condensates can be used to target signaling-driven diseases.

## Therapeutically targeting biomolecular condensates

Although the mechanisms underlying condensate formation are still being elucidated, many of their behaviors can be explained by principles derived from polymer phase separation. These principles inform a number of strategies to modulate biomolecular condensates, including approaches that are not feasible with traditional high-affinity protein interactions. Rather than relying solely on active site inhibition or protein degradation, condensate-targeting compounds can alter phase behavior, composition, or material properties. Current strategies include small molecule-mediated condensate dissolution, blocking multivalent interactions, modulating post-translational modifications, inducing regulatory or repressive condensates, and tuning condensate dynamics through physical property changes. Together, these approaches offer a versatile and mechanistically distinct toolkit for reshaping aberrant biomolecular assemblies in disease.

## Small molecule condensate dissolution

One of the most direct and widely explored therapeutic approaches involves dissolving aberrant condensates with small molecules. In the context of Wnt signaling (Fig. 1), nuclear *β*-catenin forms transcriptional condensates that drive oncogenic gene expression in colorectal cancer and other malignancies [82]. Targeting these condensates with small molecules has emerged as a promising strategy to selectively suppress Wnt-driven transcription without degrading *β*-catenin itself. One example is the small molecule YM155, which was originally developed as a survivin inhibitor and was proposed in a recent preprint to dissolve *β*-catenin condensates, although the precise molecular mechanism remains under investigation [96]. In a repurposing screen, YM155 significantly disrupted transcriptional *β*-catenin assembly and inhibited the proliferation of Wnt-driven colorectal cancer cells without affecting *β*-catenin levels [96]. These findings support the idea that condensate disruption can silence oncogenic programs without the need to eliminate the protein.

Many fusion oncoproteins acquire the ability to form aberrant nuclear condensates that drive pathological transcription [97]. Drug repurposing has also been applied in this context. A large-scale screen identified several FDA-approved compounds capable of dissolving EWS-FLI1 condensates [98]. This dissolution restored the transcriptional balance and suppressed oncogenic gene expression in Ewing sarcoma models [98]. Targeting the aberrant phase behavior of fusion oncoproteins with c-mods is a promising therapeutic strategy to reestablish normal transcriptional programs and counteract oncogenic signaling.

Small molecules that dissolve condensates also show potential in treating neurodegenerative diseases such as amyotrophic lateral sclerosis (ALS). In this context, stress granules, cytoplasmic condensates formed in response to environmental stress, fail to disassemble properly [99]. As a result, RNA-binding proteins such as TDP-43 and FUS become trapped, leading to aggregation and contributing to neuronal damage [99]. Drug repurposing efforts have identified lipoamide as a promising small molecule that prevents stress granule formation and alleviates pathology in ALS models involving FUS and TDP-43 [100]. These findings align with a growing body of research on small molecules that modulate pathological condensates in diverse contexts, including neurodegeneration, cancer, and viral infection [101]. Together, these studies support the idea that existing drugs can be repurposed to modulate the condensate state in disease, bypassing the need to develop entirely new compounds.

## Blocking multivalent interactions

**Fig. 2:**
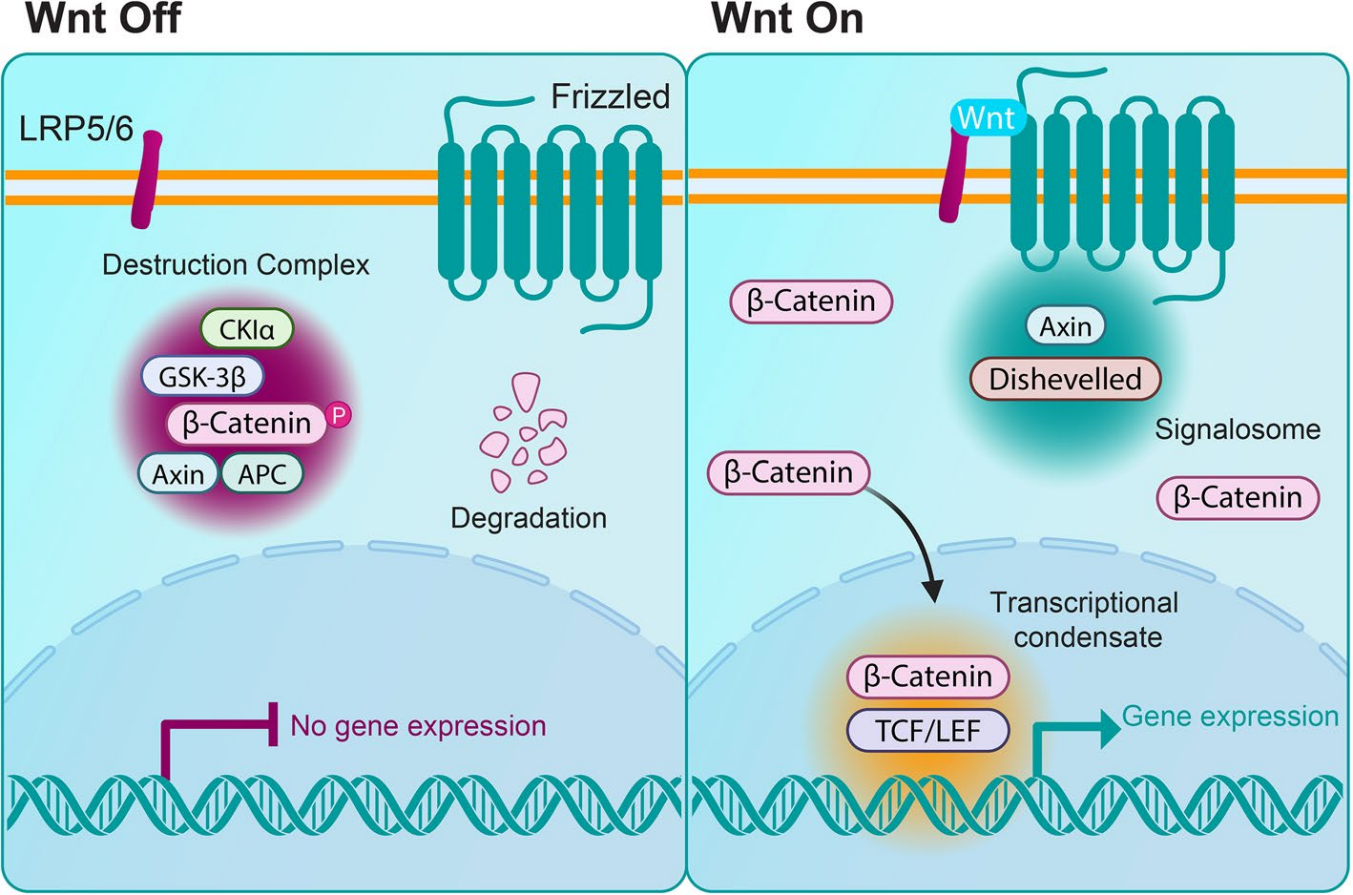
Phase separation coordinates Wnt signaling. *Left*: In the absence of Wnt ligands (Wnt Off), *β*-catenin is rapidly phosphorylated and degraded via the destruction complex, a phase-separated assembly composed of Axin, APC, GSK3, and CK1α [85, 104, 105]. This cytoplasmic condensate layer is the first level at which phase separation regulates Wnt activity. *Right*: Wnt ligand binding (Wnt On) activates Frizzled and LRP5/6, leading to Dishevelled (Dvl) recruitment and condensation at the membrane into signalosomes [106, 107], which represent a second regulatory node. These structures sequester Axin away from the destruction complex, disassembling it and leading to *β*-catenin accumulation. *β*-Catenin then translocates to the nucleus, where it forms condensates with co-activators to activate Wnt target genes [4]. This nuclear condensate assembly marks a third regulatory layer

**Fig. 3:**
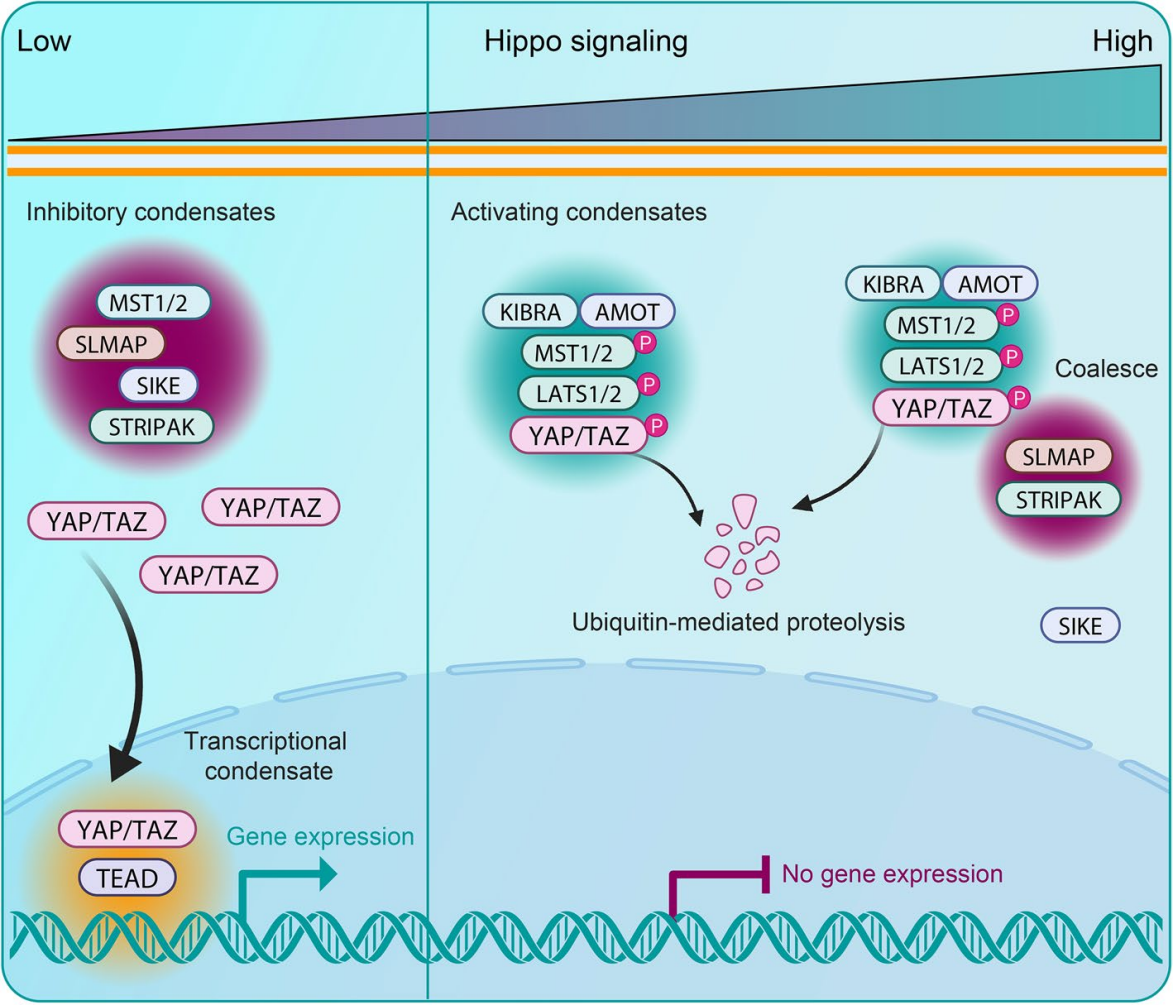
Phase separation coordinates Hippo signaling. *Left*: Inhibitory condensates formed by SLMAP, SIKE, and STRIPAK sequester and dephosphorylate MST1/2 kinases [89, 108], suppressing Hippo signaling and allowing YAP/TAZ to drive gene expression. *Right*: Cellular cues promote the formation of activating condensates driven by AMOT and KIBRA, which recruit MST1/2 and LATS1/2 kinases and facilitate the phosphorylation and degradation of YAP/TAZ. When AMOT/KIBRA and SLMAP condensates coalesce into a hybrid assembly, SIKE is excluded, allowing MST1/2 and LATS1/2 to remain active [108]. This leads to enhanced YAP/TAZ inactivation and transcriptional repression. In the nucleus, YAP/TAZ form condensates of their own. These structures act as transcriptional hubs, enriching co-activators and transcription factors such as TEAD1, MED1, and BRD4 to enhance gene expression [89, 109, 110]

Another strategy for dissolving pathological condensates involves disrupting the specific molecular interactions that drive their assembly. Short peptides designed to mimic key binding motifs can saturate interaction interfaces and prevent scaffold formation [102]. In the Wnt (Fig. 2) and Hippo (Fig. 3) pathways, for example, peptides derived from *β*-catenin and YAP/TAZ binding partners have been shown to dissolve nuclear condensates and suppress transcriptional activity by outcompeting native scaffolds for binding [102]. Extending this approach in vivo, a YAP peptide (IF1) was shown to suppress YAP-driven liver tumor growth in mouse models, supporting the disease relevance of peptide-based disruption of transcriptional condensates [103]. These studies show that engineered peptides provide a useful way to perturb condensate assembly and examine how changes in multivalent interactions influence downstream signaling. Disentangling these effects, however, is not always straightforward, since changes in condensation and transcription often occur simultaneously, but they may still arise from separate mechanisms. Approaches that control condensation with precise timing, such as light-based tools, can help separate these possibilities.

Condensate dissolution can also be achieved through synthetic RNA-based strategies. One study showed that designed RNAs can act as decoys that interfere with the phase separation of TDP-43, reversing aberrant disease-associated phenotypes in ALS patient-derived neurons [111]. By targeting the molecular interactions that drive condensation, nucleic acid-based interventions can also modulate condensate dynamics with functional consequences.

In addition to peptides and RNAs, small molecules can also directly block key interaction domains that support condensate assembly. In the context of innate immunity, recent studies have identified compounds that block cGAS condensate formation, reducing cGAS’s ability to activate downstream signaling [112]. One such example is RU.521, which disrupts the multivalent DNA binding that seeds cGAS condensates [112]. Directly targeting these assemblies may provide a way to fine-tune innate immune signaling in disease contexts.

## Formation and dissolution by modulating posttranslational modifications

In addition to dissolving condensates and inhibiting cellular processes, cells can also be induced. Inducing specific condensates can be therapeutically attractive when these assemblies support protective cellular functions or restore regulatory cellular functions, for example, by enhancing protein quality control. Although direct therapeutic strategies based on condensate induction remain limited, biological examples illustrate how promoting specific condensate assemblies can reinforce homeostatic programs. Posttranslational modification is one mechanism by which cells achieve such regulation. For example, SUMOylation promotes the assembly of promyelocytic leukemia (PML) nuclear bodies, where SUMO-modified PML acts as a scaffold to recruit additional proteins and support protein quality control [113]. This example illustrates why controlled induction of specific condensates could, in principle, be relevant in disease contexts where such regulatory mechanisms are compromised.

Posttranslational modifications can also promote condensate formation in a pathological context. In tau-related diseases, hyperphosphorylation enhances condensate formation and is associated with the accumulation of toxic assemblies in neurodegenerative diseases [114]. A similar process occurs with TDP-43, where hyperphosphorylation drives the formation of protein aggregates found in frontotemporal dementia and amyotrophic lateral sclerosis [115]. These examples demonstrate how phosphorylation can control condensate dynamics in opposite directions, suggesting that targeting specific kinases may offer a therapeutic route to dissolve pathological assemblies. In FUS, hyperphosphorylation of the low-complexity domain inhibits condensation [116]. demonstrating that posttranslational regulation can also act to limit or prevent assembly.

Modulating the phosphorylation state of condensate-forming proteins with small molecules may provide a way to change condensate behavior. Casein kinase 1δ (CK1δ) directly phosphorylates TDP-43 [117], and blocking CK1δ has been shown to inhibit this modification and to provide protective effects in neuronal models [118]. Casein kinase 2 (CK2) is another example. This kinase helps with stress granule disassembly [119]. Its activity is increased during viral infections such as SARS-CoV-2, leading to accelerated stress granule breakdown [92]. Inhibiting CK2 in this context has been suggested as a method to limit viral replication [120]. These studies illustrate how kinase activity can either dissolve or stabilize condensates by altering their posttranslational modification landscape and disrupting important interaction networks.

## Inducing regulatory condensates

While some strategies focus on dissolving harmful condensates, others aim to promote the formation of condensates composed of negative regulators. These assemblies act by reinforcing signaling checkpoints or activating degradation pathways rather than by directly targeting the disease-driving proteins themselves. By enhancing the assembly or stability of these suppressive condensates, it becomes possible to restore regulatory control over aberrant signaling without dismantling the entire pathway.

Many examples involve the Wnt/*β*-catenin pathway, where the destruction complex forms condensates that facilitate the degradation of *β*-catenin (Fig. 2). Enhancing the formation of this condensate can potently inhibit Wnt signaling. Recent studies have shown that the Gαi2 subunit of heterotrimeric G-proteins can *drive Axin into condensates*, turbo-charging the destruction complex to *clear β-catenin* [121]. In colorectal cancer cells, the expression of active Gαi2 dramatically dampened Wnt signals, whereas the removal of Gαi2 had the opposite effect [121]. The FDA-approved drug guanabenz was found to enhance this condensate-forming behavior of Gαi2, further promoting destruction complex activity and reducing *β*-catenin levels, suggesting a new therapeutic use for guanabenz in Wnt-driven cancers [121]. Encouraging beneficial condensates thus emerges as a viable route to *reinforce preexisting signaling brakes*.

In the Hippo pathway (Fig. 3), enhancing condensate formation can similarly restrain oncogenic transcription. Proteins such as AMOT and KIBRA form activating condensates in response to mechanical cues, recruiting MST1/2 and LATS1/2 to phosphorylate YAP/TAZ, thereby promoting their degradation [89, 108]. These Hippo-activating condensates can also coalesce with inhibitory STRIPAK-SIKE-SLMAP assemblies into a hybrid condensate, increasing YAP/TAZ phosphorylation and repressing downstream transcription [108]. By promoting the assembly of these negatively regulating condensates, cells can reestablish control mechanisms that are frequently lost in cancer.

Therapeutically, this mechanism offers opportunities to reinforce tumor-suppressive condensates instead of breaking down active ones. One approach may involve stabilizing AMOT or KIBRA by preventing their ubiquitination or enhancing their expression. High-content drug screening platforms could identify small molecules that enhance or sustain the co-condensation of Hippo scaffolds. These strategies support the formation of inhibitory condensate architectures and suppress YAP/TAZ signaling in cancer cells. Taken together, these findings illustrate how promoting condensation offers a viable path to control aberrant signaling.

## Inducing inert or repressive condensates

In addition to reinforcing endogenous negative regulators, condensate states that are inert or repressive by design can be induced. These assemblies act as molecular traps, sequestering key signaling proteins away from their normal interaction partners or transcriptional targets. In the Wnt pathway, the small molecule rosmanol quinone was shown to form such inert condensates. This compound promotes *β*-catenin condensation into inert cytoplasmic droplets, preventing nuclear entry and downstream transcriptional activation in liver cancer cells [43]. This *approach* neutralizes targets by driving their incorporation into nonfunctional assemblies, demonstrating a novel modality for therapeutic intervention.

Similarly, the small molecule DPTX3186 corrals *β*-catenin into inert nuclear “depot” condensates, spatially isolating it from the transcriptional machinery without dissolving active condensates [122]. This sequestration suppresses Wnt target gene activation and induces Wnt-driven cancer cell death, with low toxicity to healthy cells [122]. These findings are based on emerging, non-peer-reviewed data, and detailed mechanistic characterization is still forthcoming. This highlights an alternative mode of repression based on spatial partitioning.

Another variation involves redirecting proteins into repressive compartments. Sulforaphane (SFN), a compound found in cruciferous vegetables, has been shown to drive *β*-catenin into nuclear puncta that colocalize with repressive chromatin marks [123]. In colorectal cancer cells, these SFN-induced *β*-catenin “depots” are enriched for the transcriptional repressor PRMT5 and localize to heterochromatic regions that prevent *β*-catenin from activating Wnt target genes, although it remains to be determined whether these structures represent bona fide condensates [123]). These examples highlight how inducing inert or repressive condensates can provide a powerful alternative to traditional inhibition, functionally silencing oncogenic proteins by removing them from action without directly degrading or inactivating them.

## Altering material properties to shift dynamics

In addition to simply inducing or dissolving condensates, a more nuanced strategy involves modulating condensate material properties to shift biological function. By tuning biophysical parameters, it is possible to transform dynamic, functional condensates into inert, gel-like, or even solid-like states that no longer support disease-associated activity. In this context, the goal is to abolish condensate function by arresting molecular dynamics in a targeted manner. A recent study introduced a synthetic “kill switch” micropeptide that embeds into nuclear condensates and cross-links their internal networks, effectively freezing molecular dynamics [124]. When targeted to the nucleolus, this peptide converted normally liquid-like condensates into gel-like structures, extinguished ribosomal RNA synthesis, and induced rapid cell death. The same strategy was applied to viral systems, where retargeting the kill switch to adenoviral nuclear condensates disrupted their material state and impaired viral replication. This approach also proved effective in early zebrafish embryos, where directing the kill switch to transcriptional bodies disrupted their activity and significantly reduced microRNA production [124]. Together, these results demonstrate that condensate material properties can be rewired with modular peptides across cellular, viral, and multicellular contexts.

Similar principles appear relevant in virology, where shifting the material state of viral condensates can disrupt essential replication steps. Cyclopamine disorganizes and hardens the inclusion bodies of respiratory syncytial virus, impairing genome replication and reducing viral burden in infected mice [125]. Nucleozin drives influenza A nucleoprotein into aberrant, nonproductive aggregates that block viral propagation and protect mice from infection with Avian influenza A [126]. This ability to adjust condensate properties on demand presents new therapeutic opportunities for various diseases.

In a related approach, lipoamide, beyond its identification in drug repurposing screens, has been shown to dissolve stress granule condensates by altering their viscoelastic properties [100]. By increasing condensate liquidity and weakening intermolecular interactions, lipoamide limits the progressive hardening and fiber formation of FUS-containing condensates, a process associated with pathology in ALS models [100]. Together, these approaches demonstrate how manipulating the physical properties of condensates offers a powerful, targeted way to silence pathogenic biomolecular assemblies.

**Fig. 4:**
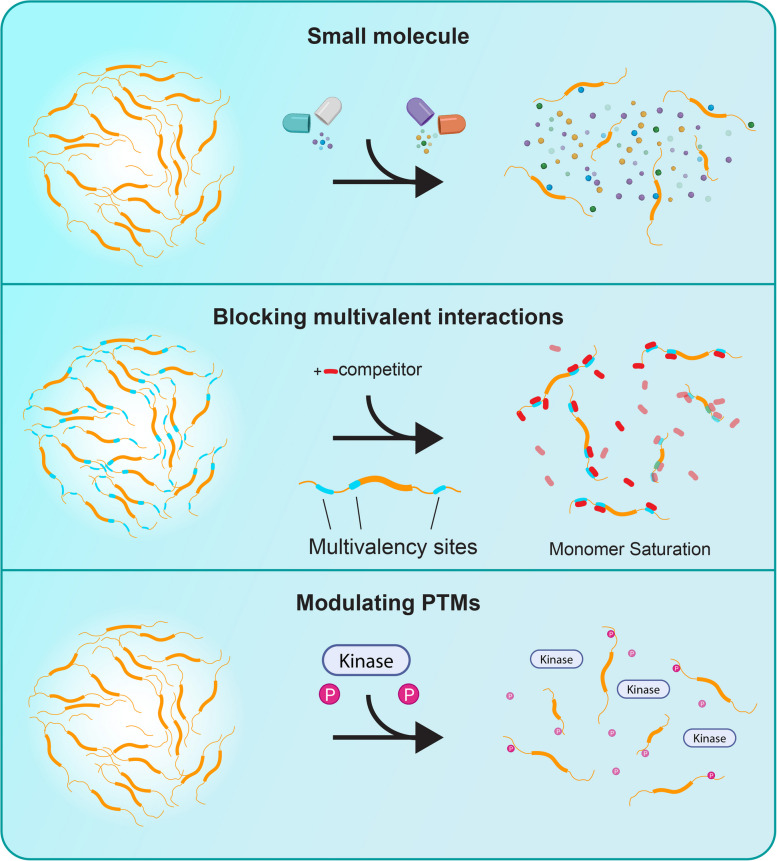
Therapeutic strategies to dissolve biomolecular condensates. *Top*: Small molecules that dissolve condensates or interfere with their assembly. *Middle*: Blocking multivalent interactions can saturate interaction domains and prevent phase separation (monomer saturation). *Bottom*: Modulating posttranslational modifications (PTMs), such as phosphorylation, alters condensate formation by shifting the interaction landscape

**Fig. 5:**
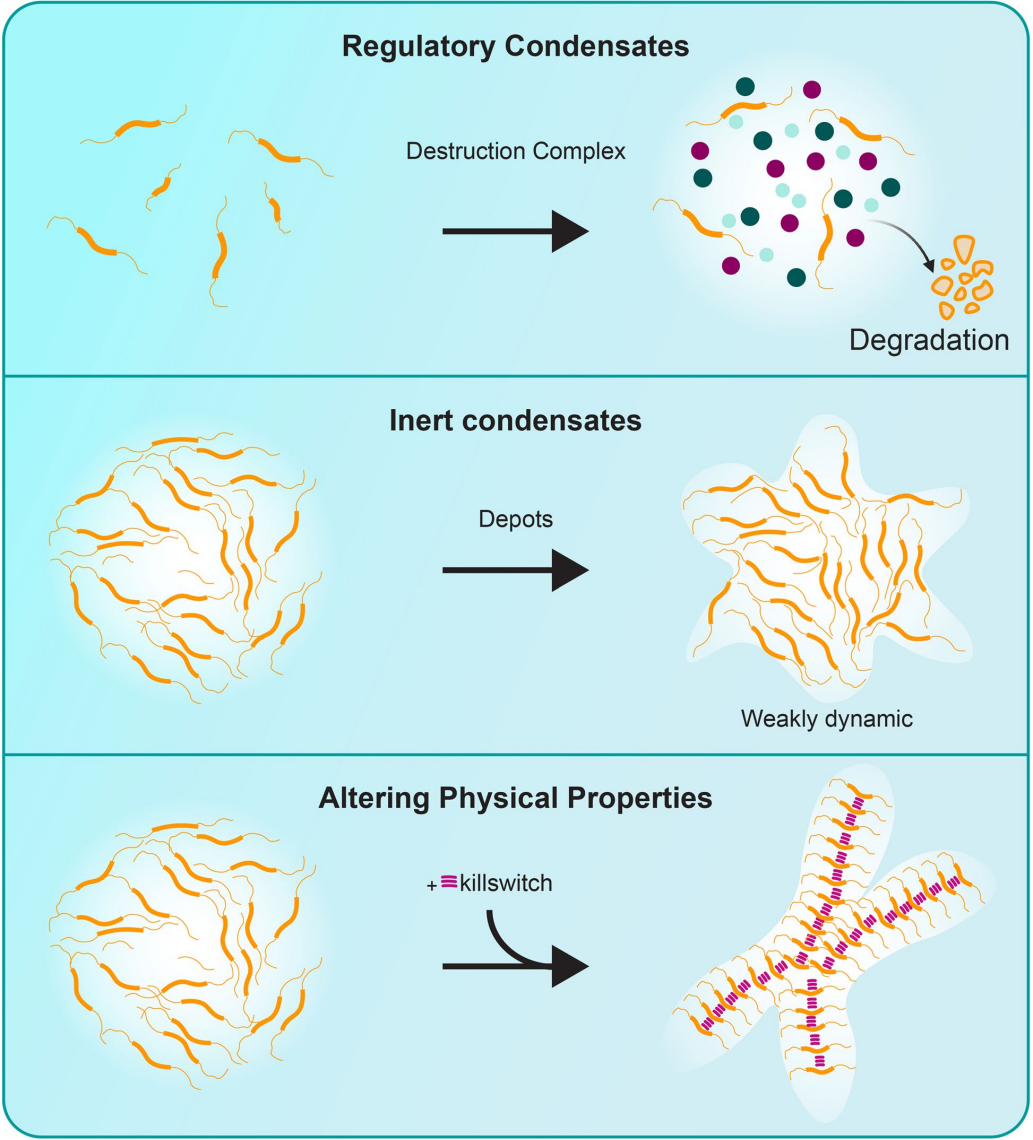
Therapeutic strategies to induce biomolecular condensates. *Top*: Activating or enhancing negative regulators promotes endogenous condensates that suppress signaling (e.g., the *β*-catenin destruction complex). *Middle*: Inducing inert or repressive condensates corrals signaling proteins into nonfunctional assemblies. *Bottom*: Altering physical properties, such as through engineered kill-switch peptides, can arrest condensate dynamics by rigidifying their internal structure

By dissolving toxic condensates, reinforcing protective condensates, or freezing their dynamics, condensate modulators promise to expand both our toolkit and the target space for treating cancer, neurodegeneration, inflammation, and infectious disease. Figures 4 and 5 provide an overview of the strategies described above, summarizing the major approaches used to dissolve or induce biomolecular condensates. With the proper biological context and molecular insight, these agents could transform our understanding of cellular regulation and therapeutic intervention alike.

## Challenges and future directions

Targeting condensates as a therapy is an exciting concept, but it comes with major challenges, most prominently achieving specificity. Numerous condensates across various pathways frequently rely on similar biophysical interactions [127–129]; thus, a universal modulator would likely produce pleiotropic effects. One possible strategy exploits unique multivalent interactions within a condensate. For example, monomer saturation via short peptides selectively perturbs the targeted Wnt or Hippo condensates without broadly disabling other phase-separated compartments [102]. Similarly, one could design c-mods that bind only to scaffold proteins unique to one condensate. An additional conceptual direction involves molecular glues, which stabilize specific protein–protein interactions and could, in principle, be adapted to induce or reinforce condensate formation in a controlled manner [130, 131]. This possibility broadens the range of chemistries that might be used to engineer condensate behavior in future applications. These examples show that precise targeting may be possible, and that condensate modulation can be harnessed as a selective and safe therapeutic approach.

A second major challenge revolves around reversibility. Permanent removal of a condensate could cause toxicity by disrupting its role during homeostasis, especially in non-diseased cells. However, persistent pathological condensates may require durable, even irreversible, disruption to be therapeutically effective. Some c-mod strategies are inherently reversible: optogenetic tools such as OptoMBP can rapidly dissolve condensates on demand and allow reformation once the light stimulus is removed [132]. Although these systems are mainly experimental, they provide clear proof of principle for temporal control. A more clinically feasible approach is the use of small molecules that can achieve similar reversibility. One example is the chemical on/off switch using the BCL6 BTB domain. BI-3802 drives reversible polymerization of a BTB-fused protein into condensates, and an antagonist (BI-3812) reverses it [133]. An additional consideration is that condensates can respond differently depending on the strength of a perturbation. Protein systems have been shown to dissolve or reform depending on the level of ionic strength applied [134]. This possibility suggests that compound concentration may also influence condensates in unexpected ways and supports the need to evaluate responses across full dose ranges. Together, these findings demonstrate that condensates can be modulated in a controlled and reversible manner.

Another major challenge lies in effectively delivering c-mods to the right cells. *Delivery* can be especially difficult for peptide- or protein-based agents. Encouragingly, the MYC inhibitor “Omomyc” can spontaneously penetrate cancer cells and inhibit MYC transcription in vivo, suggesting that engineered cell-penetrating peptides are a viable strategy [135], while lipid nanoparticle delivery systems are being engineered for nucleic acid or protein delivery to multiple tissues [136, 137].

The pharmacodynamics of condensates also raise concerns. Condensates can selectively enrich or exclude small molecules on the basis of their properties such as solubility and hydrophobicity [138, 139]. A recent study quantified the partitioning of hundreds of drugs into different condensates and reported a nearly million-fold difference in enrichment [139]. A drug may accumulate in some condensates but not others, affecting its efficacy and off-target effects. Designing c-mods with the right condensate compatibility traits is essential.

Given that condensate composition and behavior can differ greatly between various tissues, cell types, and disease contexts, all must be carefully considered. Condensates that are important for cellular function may be unintentionally affected by a c-mod designed to target pathological condensates in a different context. To minimize off-target effects, delivery strategies should prioritize specificity. Approaches such as advanced delivery vehicles conjugated to cell-type-specific ligands may provide the required spatial and contextual control.

## Shifting states and shifting strategies

Biomolecular condensates have emerged as a key concept in understanding how cellular processes are spatially and temporally controlled. In addition to their structural roles, these assemblies have been shown to actively regulate aberrant cellular programs across major diseases. In these contexts, condensates serve as functional compartments that promote gene activation and maintain signaling activity.

The ability of condensates to change their material properties has inspired a therapeutic strategy that targets these state transitions rather than solely targeting protein interactions or activity. In some cases, condensate dissolution has been shown to interrupt transcriptional output and stop disease-associated activity. In others, condensates have been pushed into gel-like or hardened states that trap proteins in nonfunctional assemblies. Such transitions provide a new layer of regulation that can be leveraged pharmacologically.

Several challenges remain, particularly in ensuring selectivity and reversibility, but as the mechanisms governing condensate dynamics are better understood, more precise tools are likely to be developed. Taken together, the modulation of condensates has the potential to impact nearly all complex molecular processes by altering their cellular organization, dramatically expanding the scope of molecular targets and compounds with therapeutic potential.

## Data Availability

No datasets were generated or analysed during the current study.
